# The perspectives of social cognitive career theory approach in current times

**DOI:** 10.3389/fpsyg.2022.1023994

**Published:** 2022-11-30

**Authors:** Danqi Wang, Xiping Liu, Haiyan Deng

**Affiliations:** ^1^Faculty of Psychology, Tianjin Normal University, Tianjin, China; ^2^School of Foreign Studies, Nanjing University of Posts and Telecommunications, Nanjing, China

**Keywords:** social cognitive career theory, boundaryless career, career intervention, assessment methods, research methods

## Introduction

Due to the rapid changes as a response to the technological innovations in the current society, the original relationship between organizations and employees has become unstable, and a new model of boundaryless career has been established (Arthur and Rousseau, 1996). The boundaryless career model, characterized by complexity, non-linearity, and unpredictability, emphasizes the influence of the environment on individuals. Among the career theories, Social Cognitive Career Theory (SCCT) first added contextual factors to the original model (Lent et al., 1994). Therefore, SCCT seems to be more applicable to the current boundaryless era.

Considering the role of individual cognitive variables (self-efficacy and outcome expectations), learning experiences, and personal interests on career development, SCCT focuses on not only environmental but also individual factors that influence one's career decision making (Lent et al., 1994). The SCCT has good applicability in school career education guidance and provides a comprehensive framework for explaining and predicting career development (Lent and Brown, 2019). The SCCT framework provides a theoretical foundation for career coaching. Compared to other career theories, SCCT offers a new perspective on guiding adolescents' interest formation, professional (career) choice, and performance, with potential for cross-cultural research (Lent et al., 2013).

Therefore, this study aims to present the advantages and challenges of SCCT and propose future research trends. The SCCT approach has the following advantages: firstly, it provides a systematic explanation for career development; secondly, it responds to the development of the times; finally, it focuses on special groups in terms of career counseling. SCCT approach also faces some challenges: lack of qualitative research methods, lack of qualitative assessment methods, and lack of intervention approach. The contribution of the study adds the number of targeted recommendations and strategies as follows: (1) Explore qualitative and quantitative research methods for SCCT; (2) Explore qualitative assessment methods for SCCT; (3) Develop multiple forms of SCCT approach.

## Social cognitive career theory

Derived from Albert Bandura's Self-Efficacy Theory and General Social Cognitive Theory (Lent, 2013), SCCT develops into a comprehensive career theory that argues that an individual's career path results from the interaction between multiple career elements since it was proposed by Lent et al. (1994). General social cognitive theory assumes that people are the product of a dynamic interaction between external environmental factors, internal subjectivity factors, as well as past and present behavior (Bandura, 1986). Self-efficacy depends on four main factors: personal performance accomplishments, vicarious learning, social persuasion, and physiological and affective states (Bandura, 1997). Drawing on Bandura's three-factor causal model, SCCT constructs a three-factor interaction model of career, in which Self-efficacy (Can I do this?), outcome expectations (what will happen if I do this?) and personal goals (how much do I want to do this?) are the three core concepts (Buthelezi et al., 2010). Rooted in learning experiences influenced by personal successes and failures experiences, vicarious learning, verbal persuasion, and affective states (Lent et al., 2017), self-efficacy and outcome expectations greatly influence one's interests, which in turn influence career choices and achievement performance (Lent et al., 1989).

Lent et al. (1994) indicated that contextual variables influence individuals' career interests and choices by shaping learning experiences in SCCT. The contextual variables of SCCT include the background contextual affordance and contextual influences proximal to choice behavior that affects career choice behavior. Among them, the background contextual affordance helps individuals to form interests and self-perceptions, while contextual influences play a role in the career decisions (Lent et al., 1994). The two types of contextual variables contain elements that overlap with each other, such as family and other social factors, these factors contribute to an individual's academic and career performance differently at different stages. More social support and specific personality traits predict more occupationally engaged behavior (Hirschi et al., 2011). The Big Five personality stands out in previous studies on personal traits. It is a significant predictor of an individual's choice behavior. Schaub and Tokar (2005) verified the relationship between Big Five personality, career learning experiences, self-efficacy, outcome expectations, and interest. The study showed that personality affects career interest directly and indirectly through career learning experiences and self-efficacy. When students present themselves as more extroverted, they seem more likely to choose a career, and when they held favorable level of conscientiousness, they experience less discomfort with decision making. Extraversion and neuroticism may influence people's interpretation of how they deal with past decisions (Penn and Lent, 2019).

The theory introduces the mechanism of the interaction of individual, behavioral and environmental factors into the career field. Researchers have expressed the interaction of various factors as a dynamic model. Social cognitive career theory initially included three interrelated models: the career interest development model, the choice-making model, and the career performance and persistence model (Lent et al., 1994), and was later expanded to include two additional models, one focusing on satisfaction and well-being model in educational and vocational settings (Lent and Brown, 2008), and the other the career self-management model, which emphasizes the process of career self-management across the lifespan (Lent and Brown, 2013).

Based on the framework of SCCT, the researcher summarized the techniques and methods of career interventions, which mainly include expanding choice options, coping with barriers, building support, goal setting and self-regulation, facilitating work performance, and promoting work satisfaction (Lent, 2013). The purpose of the SCCT intervention is to develop and modify self-efficacy related to career choices and interests, to overcome barriers related to choice and success, and to define personal goals by expanding interests and promoting choices (Barnard et al., 2008). With the theoretical framework, Miles and Naidoo (2016) examined the impact of the SCCT career program, which proves to positively affect high school students' career decision-making self-efficacy.

Chartrand and Rose (1996) were the first to suggest the application of SCCT to populations at risk for employment and occupational barriers. SCCT helps immigrant high school students to prevent dropouts, promote academic success, and foster college and career readiness through a combination of academic support and increased critical consciousness (McWhirter et al., 2019). A SCCT-based career education curriculum was designed for rural high school students which positively affected their career information about postsecondary planning and career exploration, and their planning for futures (Gibbons et al., 2019). Ali et al. (2019) evaluated the effectiveness of a sociopolitical development component in a SCCT career intervention program among rural middle school students, and found that there was limited support for the effectiveness of spd-injected SCCT interventions. Yuen et al. (2022) tested a SCCT-based career intervention program for middle school students with mild special educational needs and found it impacted the students' career, personal and social development self-efficacy, and acquisition of a sense of meaning in life. Silva et al. (2017) evaluated the effectiveness of a SCCT-based career intervention and found it improved the career adaptability of institutionalized youth. Also, Glessner et al. (2017) found that a workshop on the online Florida CHOICES program and a campus visit increased semirural school student career and college self-efficacy. The study supported the practice of engagement in a virtual- and community-based intervention as a teaching strategy.

## Advantages of social cognitive career theory

### Provide a systematic explanation for career development

SCCT values the role of psychological factors (interests, abilities, values), social factors (e.g., socioeconomic status, gender, race), and economic factors (e.g., employment opportunities, training opportunities, etc.) (Long et al., 2002). By doing so, SCCT attempts to create an integrated framework that overcomes the limitations of traditional theory that separates psychological, social, and economic factors. SCCT more systematically elucidates how the interaction between core cognitive, personal, and environmental variables contributes to an individual's career development.

### Respond to the development of the times

Lent et al. (1994) constructed the SCCT framework, which argues that interest arises from self-efficacy and outcome expectations. Self-efficacy and outcome expectations change dynamically as learning experiences change, and a developmental perspective is used to view career development. The theory emphasizes that goal selection is dynamic and that environmental factors influence goal setting, in line with the current boundaryless era.

Traditional career theories ignore the social environment factors, emphasize the matching of personality traits with careers, and ignore the influence of the environment on career development. SCCT emphasizes individual and environmental changes and views career choice as a relatively dynamic system as time changes, which proves to be more adaptive to the contemporary society than traditional career theories.

### Focus on special groups of career counseling

SCCT proposes that environmental factors impact individual career development and directly influence the formation of learning experiences. It extends to special groups and offers possibilities and strategies for career counseling for special groups. Much related research has focused on special groups, such as individuals with serious mental health disorders (Fabian, 2000), institutionalized youth (Silva et al., 2017), immigrant high school students (McWhirter et al., 2019), rural school students (Ali et al., 2019; Gibbons et al., 2019), and secondary school students with mild special educational needs (Yuen et al., 2022).

## Challenges to social cognitive career theory

### Lack of qualitative research methods for SCCT

Previous studies tend to use quantitative methods to validate the SCCT framework (Lent et al., 2017; Penn and Lent, 2019), while few studies explore the career selection process adopting qualitative research approaches. At the same time, SCCT focuses on self-efficacy, outcome expectation, and learning experience when explaining career choice and development. Among these factors, which one plays the most important role, and to what degree they can predict career performance is unclear. This impedes the application of SCCT to the educational settings.

### Lack of qualitative assessment methods for SCCT

SCCT emphasizes the role of learning experiences, environmental factors, and interests, which consequently leads to the ambiguity of the evaluation criteria, especially the qualitative evaluation methods. Some researchers conducted a quantitative assessment method by Career Decision Self-Efficacy Questionnaire (Miles and Naidoo, 2016; Glessner et al., 2017).

Among the few studies that used qualitative assessments, one study conducted interviews with five teachers and social workers (Yuen et al., 2022). There is no uniform criteria for qualitative evaluation. However, qualitative assessment methods may be complicated, especially for special groups.

### Lack of intervention approach for SCCT

Due to the COVID-19 pandemic, it was difficult to conduct a follow-up survey to identify the long-term effects of the SCCT Project (Yuen et al., 2022). On the other hand, most of the studies on SCCT have focused on specific groups, and it is difficult to expand the findings to the general population.

However, researchers proposed a Multi-Tiered System of Support for students: the first tier of the framework is the curriculum to promote mental health for all students. The second is group counseling for some students, which meets the developmental needs to prevent problems and cope with the confusion of students' growth. The third is the intervention for individual students to address their special mental health problems (Fang et al., 2014; Sulkowski and Michael, 2014). The SCCT interventions mostly take the form of the workshop (Ali et al., 2019; McWhirter et al., 2019), and individual counseling may be more appropriate for special groups. In summary, multiple forms of SCCT interventions need to be developed.

## Discussion

### Explore qualitative and quantitative research methods for SCCT

Sheu et al. (2010) used meta-analytic path analyses to synthesize data from 1981 to 2008 and found that both self-efficacy and outcome expectations are significant predictors of interests, and that interests partially mediate the effect of self-efficacy and outcome expectations on choice goals. However, inconsistent findings were shown by Garriott et al. (2014), which examined the relations of high school students' learning experiences, self-efficacy, outcome expectations, and interests. The study showed that self-efficacy significantly predicted interests, but did not predict outcome expectations. In addition, outcome expectations did not predict interests (Garriott et al., 2014). Although SCCT provides an important theoretical basis for explaining and predicting academics and careers, research on the relationship between learning experiences, self-efficacy, outcome expectations, and interests has not yet yielded consistent findings.

Qualitative and quantitative research methods deserve to be combined in the field of career (Pan and Sun, 2018). As previous studies on SCCT mainly used quantitative methods, future research should combine qualitative and quantitative methods to explore the relationship between the different variables of SCCT.

### Explore qualitative assessment methods for SCCT

Lent and Brown (2020) systematically introduced a three-factor (content-process-context, CPC) assessment model to determine what issues exist and how to prioritize them in counseling. The use of assessments early in the counseling can gauge the need in terms of decision-making processes, contextual barriers, and/or choice of content options. Future studies on the evaluation of SCCT can be conducted on the following three factors. (1) Content: Assessment can be based on the variables included in the SCCT, namely interest, outcome expectancy, self-efficacy, learning experience. For example, for learning experiences, assessments are made in terms of achievement events, alternative experiences, emotional state, and social encouragement. (2) Process: To identify difficulties with the decision-making process, such as choice anxiety, Interpersonal conflict, and low decision-making self-efficacy. (3) Context: To assess critical environmental characteristics that can aid or impede selection, such as contextual supports, contextual barriers, and factors related to the selection. In terms of sequencing the assessment, it may be careful to collect decision-making process and contextual information either prior to or along with content data, as a rush to evaluate interests may ignore personal or contextual factors affecting responses to the assessment instrument, the understanding of results, or the willing to engage in exploration or to make a decision (Lent and Brown, 2020).

### Develop multiple forms of the SCCT approach

With the development of the Internet, the online career network system can effectively offset the shortcomings of traditional intervention. The computer is accessible anytime and anywhere without physical contact, so the problems in the conventional intervention model can be solved online. With the development of computer networks, career intervention has evolved into an online format. SCCT is the framework for developing mobile phone-based intervention (Ho et al., 2020).

As an important research direction, SCCT intervention can be designed around career guidance and counseling in school. In addition, the primary forms of career intervention are career class, group career counseling, workshop, computer network system, and individual counseling (Whiston et al., 2017). Regarding the student development guidance model, the school career development is designed on three levels. The first level of career intervention is open to all students and is designed with a practical career curriculum according to the psychological developmental characteristics. On the second level, students with specific needs, identified on the first level, are chosen for group counseling. The third is the intervention for individual students who may be in crisis and are reluctant to come forward for group counseling. To sum up, the school career intervention system is based on the three levels of career development.

Future research should develop multiple interventions and evaluate the effects of specific career interventions (e.g., self-service, computer network systems, career counseling, etc.) on different groups so as to develop systems for SCCT interventions.

## Conclusion

Based on General Social Cognitive Theory and Self-Efficacy Theory, SCCT is an effective form of career counseling in the boundaryless era. While SCCT has strong implications for career counseling and education, it also has deficiencies in research methods, evaluation methods and intervention methods. Therefore, this study offers some suggestions on how to address these challenges: Firstly, future research should combine qualitative and quantitative methods to explore the relationship between the variables of SCCT. Secondly, future research need to explore qualitative assessment methods for SCCT. Finally, researchers are hoped to develop diversified intervention methods for SCCT so that students with different needs all can obtain career development.

## Author contributions

DW and XL contributed to design of the study. DW wrote the manuscript. DW and HD modified the manuscript. All authors contributed to the article and approved the submitted version.

## Conflict of interest

The authors declare that the research was conducted in the absence of any commercial or financial relationships that could be construed as a potential conflict of interest.

## Publisher's note

All claims expressed in this article are solely those of the authors and do not necessarily represent those of their affiliated organizations, or those of the publisher, the editors and the reviewers. Any product that may be evaluated in this article, or claim that may be made by its manufacturer, is not guaranteed or endorsed by the publisher.
